# Blood Vessel-Targeted Therapy in Colorectal Cancer: Current Strategies and Future Perspectives

**DOI:** 10.3390/cancers16050890

**Published:** 2024-02-22

**Authors:** Anne Jacobsen, Jürgen Siebler, Robert Grützmann, Michael Stürzl, Elisabeth Naschberger

**Affiliations:** 1 Division of Molecular and Experimental Surgery, Translational Research Center, Universitätsklinikum Erlangen, Friedrich-Alexander-Universität Erlangen-Nürnberg (FAU), Kussmaulallee 12, D-91054 Erlangen, Germany; anne.jacobsen@uk-erlangen.de (A.J.); michael.stuerzl@uk-erlangen.de (M.S.); 2 Comprehensive Cancer Center Erlangen-EMN (CCC ER-EMN), D-91054 Erlangen, Germany; juergen.siebler@uk-erlangen.de (J.S.); robert.gruetzmann@uk-erlangen.de (R.G.); 3 Department of General and Visceral Surgery, Universitätsklinikum Erlangen, Friedrich-Alexander-University Erlangen-Nürnberg (FAU), D-91054 Erlangen, Germany; 4 Department of Medicine 1—Gastroenterology, Universitätsklinikum Erlangen, Friedrich-Alexander-University Erlangen-Nürnberg (FAU), D-91054 Erlangen, Germany

**Keywords:** antiangiogenic treatment, cancer, colorectal cancer, endothelial cells, tumor microenvironment, bevacizumab, ramucirumab, aflibercept, regorafenib, fruquintinib, angiocrine, vasculature, vascular heterogeneity

## Abstract

**Simple Summary:**

This review summarizes the history and current clinical applications of antiangiogenic treatment. It specifically discusses current challenges of the treatment and opportunities for optimization, including normalization of the tumor vasculature, modulation of milieu-dependent heterogeneity of the vasculature, and targeting of angiocrine protein functions.

**Abstract:**

The vasculature is a key player and regulatory component in the multicellular microenvironment of solid tumors and, consequently, a therapeutic target. In colorectal carcinoma (CRC), antiangiogenic treatment was approved almost 20 years ago, but there are still no valid predictors of response. In addition, treatment resistance has become a problem. Vascular heterogeneity and plasticity due to species-, organ-, and milieu-dependent phenotypic and functional differences of blood vascular cells reduced the hope of being able to apply a standard approach of antiangiogenic therapy to all patients. In addition, the pathological vasculature in CRC is characterized by heterogeneous perfusion, impaired barrier function, immunosuppressive endothelial cell anergy, and metabolic competition-induced microenvironmental stress. Only recently, angiocrine proteins have been identified that are specifically released from vascular cells and can regulate tumor initiation and progression in an autocrine and paracrine manner. In this review, we summarize the history and current strategies for applying antiangiogenic treatment and discuss the associated challenges and opportunities, including normalizing the tumor vasculature, modulating milieu-dependent vascular heterogeneity, and targeting functions of angiocrine proteins. These new strategies could open perspectives for future vascular-targeted and patient-tailored therapy selection in CRC.

## 1. History and Development of Antiangiogenic Treatment for Cancer

Colorectal cancer (CRC) is the third most common cancer and accounts for approximately 10% of cancer cases worldwide [1]. CRC incidence rates remain high in highly developed countries such as Canada and Northern Europe and are rising rapidly in many less developed countries, particularly in Eastern Europe, Asia and South America [2]. The established risk factors for CRC include high intake of processed meats and low intake of fruits and vegetables, a sedentary lifestyle, obesity, smoking, and excessive alcohol consumption [1]. The introduction of population-based screening in a growing number of countries likely contributed to decreasing mortality rates in some regions [2]. Since the 1990s, despite an overall downward trend, particularly in high-income countries, there has been an increase in digestive tract cancers in adults under the age of 50 [3]. Despite these recent observations, CRC remains significantly more common in older people. Considering the steadily increasing global life expectancy at birth, a doubling of the incidence of CRC in old world regions by 2035 has been predicted [2].

Although the prognosis for CRC has improved in recent decades, this disease is still responsible for 880,000 deaths globally [2]. Approximately 15–30% of patients present with metastases at the time of diagnosis, and more than 20% of patients with initially localized disease will develop metastases over time [4]. These high numbers require continued intense and relentless efforts to combat the disease. The most urgent targets for improvement are the expansion of prescreening programs, education about a tumor-preventing lifestyle, the availability of healthy food to the global population and improved forms of therapy in association with specific approaches to predetermine therapy responses.

In accordance with the clinical need for improvement in treatment regimens, we will focus here on the status of CRC therapy, specifically by analyzing the role of the vascular system as a therapeutic target. With the appreciation of the important role of the microenvironment in carcinogenesis approximately 15 years ago, it became clear that not only tumor cells alone but also the interplay of tumor cells with the different cell types in the surrounding stroma mediated by many different cytokines and growth factors is a paramount denominator of tumor progression and therapeutic responses [5]. In this framework, it is highly remarkable that more than 50 years ago, Judah Folkman had already recognized the importance of the vasculature as a stromal-derived component for tumor therapy. His hypothesis was that vessels are needed for the delivery of nutrients to tumor cells and that blocking vessel growth into tumors may consequently reduce tumor progression [6]. In comparison to tumor cell-directed cancer therapy, this approach is thought to have several advantages, including (i) reduced resistance achieved by targeting genetically stable tumor vessel endothelial cells (TECs) instead of tumor cells, where genetic instability is an important driver of resistance. (ii) Furthermore, the endothelium is considered to be easily accessible to drugs applied through the blood circulation. (iii) Finally, it was shown that approximately one endothelial cell delivers nutrients to up to 100 tumor cells, and accordingly, amplification effects are expected in endothelial cell-directed therapy [7].

These findings initiated a series of fascinating experimental approaches in animal models, which convincingly supported the important role of the vascular system in tumor therapy. All of these findings have been comprehensively reviewed in the literature [8,9]. Consequently, only some of the most important results are highlighted in the following section.

One of the first requirements for antiangiogenic therapy was the availability of the respective inhibitors. In the first step, these substances directly inhibit endothelial cell proliferation or migration. Among these substances was fumagillin, which was first isolated from *Aspergillus fumigatus* in 1949 as an antiphage agent [10,11]. Fumagillin was shown to be an antiangiogenic agent when Folkman’s coworker revealed that it inhibited capillary endothelial cell proliferation in *Aspergillus fumigatus*-contaminated cell cultures [12]. To reduce its nonspecific toxicity, derivatives of fumagillin were synthesized, and these substances significantly inhibited tumor growth in preclinical experimental tumor models [12]. A further key development was based on two important points: first, the observation that the growth of metastases dramatically increased after surgical removal of the primary tumor in certain rodent carcinoma models; second, the hypothesis that inhibitors of angiogenesis may be enriched in the primary tumors but trumped by stimulators; and that this balance may be shifted distantly in the circulation when inhibitors are more stable and stimulators are rapidly cleared [9]. These conditions specifically inhibit angiogenesis, which is needed for distant metastasis formation in the presence of the primary tumor. Based on these considerations, two angiogenesis inhibitors that are released in proteolytically active primary tumors as cleavage products of other proteins were identified. These inhibitors were named angiostatin, a cleavage product of the blood protein plasminogen involved in fibrolyis, and endostatin, a cleavage product of the extracellular matrix protein collagen XVIII [13,14]. Both proteins specifically inhibited endothelial cell proliferation but had no effect on resting endothelial cells or other cell types [9,13,14]. Moreover, endostatin and angiostatin strongly inhibited the growth of many different tumors, including breast, colorectal and lung cancer, in different mouse models [15]. Most importantly, for endostatin, this drug did not lead to acquired drug resistance after several cycles of treatment or tumor regrowth during phases where treatment ceased [16]. These results led to great euphoria and high hopes in researchers, medical doctors and patient populations with respect to antiangiogenic cancer therapy in humans. This culminated when *The New York Times* headlined these findings in 1998 and cited the codiscoverer of the DNA structure and Nobel laureate James D. Watson with the sentence “Judah Folkman is going to cure cancer in two years” [17]. This expression is often used to demonstrate excitement in the field but was quickly contradicted by Watson himself, who stated that he was misquoted and instead referred to the urgent need for clinical trials, which would show within the year whether the substances are effective [18]. Folkman’s statements were more focused on the actual facts when he was cited: “If you have cancer and you are a mouse, we can take good care of you” [17]. In fact, in the effort to translate the preclinical results to clinical therapy, severe pitfalls arose, and altogether, there was less excitement when these substances were examined in clinical studies. It took several years until 2004 when antiangiogenic therapy was successfully applied to a human cancer, namely, CRC, for the first time [19]. In the following paragraphs, we will specifically discuss the present standing of antiangiogenic therapy in CRC and summarize putative reasons for therapy failure in humans as well as putative perspectives.

## 2. Clinical Application of Antiangiogenic Treatment in Colorectal Cancer

**Figure 1:**
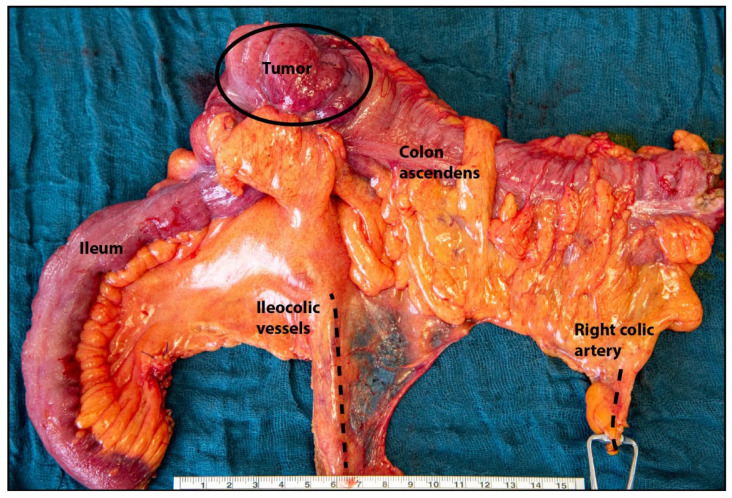
The tumor-related vascular structure and hierarchy determine the surgical resection strategy used for colorectal cancer. Surgical preparation after right hemicolectomy with complete mesocolic excision because of cecal carcinoma (circle). Central ligation of the ileocolic vessels (artery and vein) and the right colic artery (dashed lines) ensures resection of the regional lymph nodes, which is well known to improve survival.

The vasculature plays an essential role in CRC therapy. First, angiogenesis is the target of antiangiogenic therapy, as explained above. Second, the vasculature also determines the extent of surgical resection of CRC (Figure 1). Two groups of antiangiogenic drugs are currently used to treat metastatic CRC (mCRC): monoclonal antibodies and small molecules, specifically tyrosine kinase inhibitors (TKIs) [20] (Table 1).

### 2.1. Monoclonal Antibodies

Currently, clinically used monoclonal antibodies block the VEGF-VEGFR2 axis and, accordingly, the activation of VEGF signaling pathways. Bevacizumab, the first approved antiangiogenic drug, binds to VEGF-A and prevents its binding to the corresponding receptors [27]. Aflibercept is a soluble VEGF receptor that also captures VEGF before it can bind to the respective cellular receptors. Ramucirumab binds directly to VEGFR2, thereby inhibiting its activation [20]. All three antibodies are used globally in combination with chemotherapy in standard second-line therapy for unresectable CRC. However, only bevacizumab is recommended in first-line setting [4,28,29]. Toxicity and adverse effects, including hypertension, proteinuria, hemorrhage, GI perforation, wound complications, and thromboembolic events, are mostly modest and manageable [30,31,32,33].

#### 2.1.1. Bevacizumab

Bevacizumab was the first antiangiogenic drug approved for clinical application, and it is still the most widely used [27]. In mCRC, bevacizumab was established in first and later lines of therapy in combination with chemotherapy, as monotherapy has no relevant impact in mCRC [20]. In the first clinical trials, bevacizumab seemed to improve the response rate (RR), progression-free survival (PFS), and overall survival (OS) in combination with chemotherapy. Kabbinavar et al. [34] reported a dose-dependent positive effect of bevacizumab in combination with 5-FU/leucovorin on the RR, PFS and OS in a phase II trial in patients with mCRC. Hurwitz et al. [19] also reported better outcomes for all three parameters for patients treated with bevacizumab in combination with bolus 5-FU/leucovorin/irinotecan than for patients treated with the same chemotherapeutic regimen plus placebo in first-line therapy for untreated mCRC. These outcomes resulted in the approval of bevacizumab for the first-line treatment of metastatic colorectal disease.

In the following years, different combinations of bevacizumab and chemotherapeutic regimens were tested in many clinical trials. Although some of the studies reported improved PFS, the OS did not improve by adding bevacizumab [21,35,36,37,38].

Bevacizumab in first-line therapy seems to improve both OS and PFS when combined with fluoropyrimidine monotherapy, for example for patients with reduced general health, but only PFS when combined with commonly recommended combined chemotherapies based on infusional 5-FU (FOLFOX, FOLFIRI) [39].

Several clinical trials have compared bevacizumab to anti-EGFR agents, such as cetuximab or panitumumab, in first-line therapies. Heinemann et al. compared FOLFIRI/cetuximab versus FOLFIRI/bevacizumab as first-line treatment for patients with KRAS wild-type (wt) mCRC in a randomized phase III trial (FIRE-3) and reported a significantly prolonged OS in the cetuximab group (28.7 vs. 25.0 months in the bevacizumab group), although RR and PFS did not significantly differ [40]. Venook et al. conducted a similar trial (CALGB/SWOG 80405) to test either FOLFIRI or FOLFOX plus cetuximab or bevacizumab and found no differences in OS, PFS and RR between bevacizumab and cetuximab [41]. In the phase II PEAK study, when comparing FOLFOX plus panitumumab or bevacizumab as first-line therapy in patients with unresectable KRAS-wt mCRC, a prolonged OS and a similar PFS were found for the panitumumab group with KRAS-wt exon 2 [42]. Today, it is well known that right-sided and left-sided CRC differ clinically and molecularly, so sidedness is essential for clinical trials and therapeutic decisions. Holch et al. analyzed the primary tumor location in relation to the response to anti-EGFR therapy versus anti-VEGFR therapy in a meta-analysis of the three abovementioned studies (FIRE-3, CALGB/SWOG 80405, PEAK). The authors concluded that patients with left-sided RAS-wt mCRC benefit from treatment with an anti-EGFR antibody, whereas bevacizumab should be preferred for right-sided mCRC [43]. Sidedness was also addressed in a retrospective subgroup analysis of the two pivotal first-line bevacizumab trials of Hurwitz et al. (2004) [19] and Saltz et al. (2008) [37] mentioned above. This retrospective analysis revealed that bevacizumab had an effect independent of tumor sidedness in mCRC [44].

Currently, bevacizumab is regularly used in combination with different first-line chemotherapies for the treatment of mCRC. Its combination with FOLFOX, FOLFIRI or the triplet FOLFOXIRI is recommended for right-sided RAS- and BRAF-wt mCRC but also for RAS-mut and BRAF-mut mCRC, independent of the sidedness [4,28], although its efficacy, in particular in combination with potent chemotherapies as FOLFOX, FOLFIRI or FOLFOXIRI is still unclear.

The efficacy of bevacizumab was also analyzed for maintenance and second-line treatment. The CAIRO3 trial demonstrated capecitabine plus bevacizumab, and the AIO 0207 trial demonstrated fluoropyrimidine plus bevacizumab as preferable options for maintenance therapy in mCRC [45]. In second-line treatment, the combination of chemotherapy and bevacizumab compared to chemotherapy alone improved OS and PFS in different phase III trials, although the absolute benefit was only 1–2 months in terms of the median OS [46].

Today, bevacizumab is regularly used in combination with first- and second-line chemotherapy as well as maintenance therapy in the treatment of mCRC. Moreover, starting in 2023, bevacizumab has been used in last-line therapy in combination with trifluridine-tipiracil, as the SUNLIGHT trial showed a relevant improvement in OS (10.8 versus 7.5 months) and PFS (5.6 versus 2.4 months) for patients treated with trifluridine-tipiracil plus bevacizumab compared to those treated with trifluridine-tipiracil alone [47].

#### 2.1.2. Ramucirumab

Ramucirumab is a human immunoglobulin G1 (IgG 1) monoclonal antibody that blocks VEGF receptor 2 (VEGFR-2), thereby preventing its activation [24]. In the RAISE study, a phase III clinical trial, ramucirumab was tested in a second-line setting with FOLFIRI in patients with mCRC who had disease progression during or within six months after first-line therapy with bevacizumab, oxaliplatin and a fluoropyrimidine. Patients treated with ramucirumab/FOLFIRI had a significantly longer OS (13.3 months) than patients treated with placebo/FOLFIRI (11.7 months) and a significantly longer PFS [24].

#### 2.1.3. Aflibercept

Aflibercept is a fusion protein of the VEGF-binding domain of VEGFR1 and VEGFR2 with an Fc fragment of a human IgG1 antibody. This protein is a high-affinity ligand trap for VEGFA, VEGFB, and placental growth factor (PlGF), thereby preventing the binding of these proteins to VEGFR [23]. In a phase III clinical trial, patients treated with aflibercept in combination with FOLFIRI had significantly better OS (13.5 vs. 12.6 months) and PFS (6.9 vs. 4.7 months) than did patients treated with FOLFIRI/placebo in second-line therapy after previous treatment with oxaliplatin [23].

It is unclear which of the three antibodies should be preferred in the second-line treatment of mCRC [48]. To address this question, Hashimoto et al. initiated the ongoing prospective randomized phase II clinical trial (JCOG2004) to compare bevacizumab with ramucirumab and aflibercept, each in combination with FOLFIRI, in second-line treatment for unresectable CRC after first-line therapy with fluoropyrimidine and oxaliplatin [49].

### 2.2. Tyrosine Kinase Inhibitors

Antiangiogenic tyrosine kinase inhibitors (TKIs) are small molecules that traditionally affect a wide range of tyrosine and serine-threonine kinases in addition to the intended VEGFR signaling pathway [50]. Due to this low selectivity, TKIs often cause serious toxicity, making their clinical use challenging, particularly in combination with chemotherapy [51]. Moreover, the combination of TKIs with chemotherapy in mCRC patients has been disappointing [20]. In monotherapy, the typical adverse effects of the TKIs regorafenib and fruquintinib, used in mCRC therapy, include hypertension, hand-foot skin reaction, diarrhea, fatigue and dysphonia [25,52].

#### 2.2.1. Regorafenib

Until recently, the only TKI used in the clinical treatment of mCRC was regorafenib, an oral multikinase inhibitor with activity against VEGFR-2, VEGFR-3, TIE-2, platelet-derived growth factor receptor (PDGFR), fibroblast growth factor receptor (FGFR), rearranged during transfection (RET) and c-Kit, as well as a signal transduction inhibitor of the RAF/MEK/ERK pathway [33]. Regorafenib had a statistically significant, but only moderate, effect on OS (6.4 vs. 5.0 months) as monotherapy for chemorefractory patients with mCRC compared to placebo in the phase III multicenter CORRECT trial [25].

##### 2.2.2. Fruquintinib

Fruquintinib is a new antiangiogenic TKI that targets VEGFR. It is a small molecule that is orally applied and, in contrast to regorafenib, exhibits high selectivity for VEGFR-1, -2 and -3 [52]. Its effectiveness in mCRC was evaluated in the pivotal FRESCO trial, a randomized, double-blinded, multicenter phase III clinical trial in China that compared fruquintinib monotherapy versus placebo in patients with mCRC and progression after two lines of chemotherapy without VEGFR inhibitors. Median overall survival (9.3 vs. 6.6 months) and PFS (3.7 vs. 1.8 months) were significantly better in the fruquintinib group than in the placebo group [53]. These results led to the approval of fruquintinib for third- or later-line therapy for mCRC in China [54]. The authors state that the results may not be applicable to the Western population, as the standard treatment for mCRC in China does not include anti-VEGF therapy in prior therapy lines [53]. This phenomenon has been addressed in the global FRESCO-2 trial (NCT04322539), which included almost 700 patients from the United States, Europe, Japan and Australia [26]. The results showed a promising effect of fruquintinib in the treatment of patients with advanced, chemotherapy-refractory mCRC, with an OS of 7.4 months in the fruquintinib group versus 4.8 months in the placebo group [26]. These outcomes resulted in the recent FDA approval of fruquintinib for previously treated mCRC in November 2023.

Several additional monoclonal antibodies, a peptibody and many TKIs that target tumor angiogenesis through multiple pathways have been tested in mCRC patients in recent decades. Unfortunately, most of these agents showed no relevant efficacy in therapy of mCRC or had unfavorable toxicity. An overview of some of these regimens that have reached randomized phase II/III clinical trials but did not obtain approval for clinical application in mCRC is given in Table 2. Notably, some of those regimens are still under evaluation in combination with other therapies.

## 3. Challenges in Antiangiogenic Treatment of CRC

To discuss the current challenges of antiangiogenic treatment in CRC, we need to rethink the original aims of the treatment. Initially, antiangiogenic treatment was thought to completely cut off the tumor from its blood supply to induce starvation of the tumor cells, thereby stopping tumor growth and inducing tumor cell death and tumor regression. However, it soon became evident that a superior therapeutic effect is observed by “normalization” of the tumor vasculature [51]. In the context of tumor vessel normalization, also defined as vessel pruning and regression, oxygenation and perfusion of the tumor improve in association with a reduction in tumor vessel size and tortuosity [74]. This results in the restoration of vascular maturation, increased capacity to sustain tissue pressure and normalization of the basement membrane [75]. This approach ultimately allows improved delivery and efficacy of chemotherapeutic drugs given in combination with antiangiogenic treatment [74]. This concept is supported by the observation that cytotoxic therapy results in a better outcome within the window of tumor vessel normalization than did cytotoxic therapy before or after [76]. Unfortunately, as detailed in the previous section, antiangiogenic treatment combined with chemotherapy causes only mild increases in survival, low response rates and moderate efficacy [19]. Notably, even partial progression of disease under treatment or treatment resistance occurs together with a lack of bio-markers for stratifying patients [51,77,78,79]. Based on these issues, we discuss below the predominant challenges in the antiangiogenic treatment of colorectal cancer.

### 3.1. Dosing and Timing of Antiangiogenic Treatment

The dosing and timing of the current treatment schedule are parameters that impact the outcome of antiangiogenic treatment. This issue was addressed by comparing conventional schedules of chemotherapy with the maximum tolerated dose (MTD) combined with antiangiogenic treatment to alternative metronomic dosing schedules. Metronomic treatment regimens are characterized by the administration of lower doses than the MTD but at a greater frequency. The application of such metronomic schedules showed an increased clinical benefit, particularly in the metastatic disease setting, together with the advantage of lower overall toxicity [80]. This finding has the potential to update the current application schemes accordingly in the future. However, it should be noted that the high frequency of drug administration is challenging for patients, and reports of a lacking advantage of this metronomic therapy schedule also exist [81]. Moreover, overdosing is known to hamper the efficacy of antiangiogenic treatment, as it has been reported that low-dose anti-VEGF treatment sensitizes patients more efficiently to PD-1 blockade than does the conventional dose [82].

### 3.2. Combination of Antiangiogenic Treatment with Alternative Drugs

Many studies currently in progress aim to overcome the limitations of antiangiogenic treatment by combining antiangiogenic agents with alternative regimens, such as novel immunomodulatory drugs. The most common treatment regimens are anti-VEGF therapy in combination with PD-1 blockade, for example, the addition of atezolizumab (an anti-PD-L1 antibody) to capecitabine and bevacizumab [83]. Additionally, the combination of angiopoietin-2 (ANG2) blockade and VEGF plus immunotherapy is currently being investigated and has shown promising results [82,84,85,86]. Interestingly, in this context, triple blockade of PD-1, ANG2 and VEGF resulted in increased CTL levels and global tumor vessel normalization, which was greatest in the triple therapy scheme [86]. Accordingly, the combination of antiangiogenic treatment with immunomodulatory drugs has great additional potential by overcoming milieu-dependent immunosuppressive functions and further increasing therapeutic efficacy by fostering vessel normalization [86,87]. Notably, efforts are also underway to optimize the combination schedules of different drugs by developing algorithms that predict optimal low-dose drug combinations to improve the outcome of antiangiogenic treatment, and these algorithms have shown beneficial effects [85,88].

### 3.3. Heterogeneity of ECs According to Vessel Type, Organ, Disease, Patient, EC Hierarchy and Activation State

**Figure 2:**
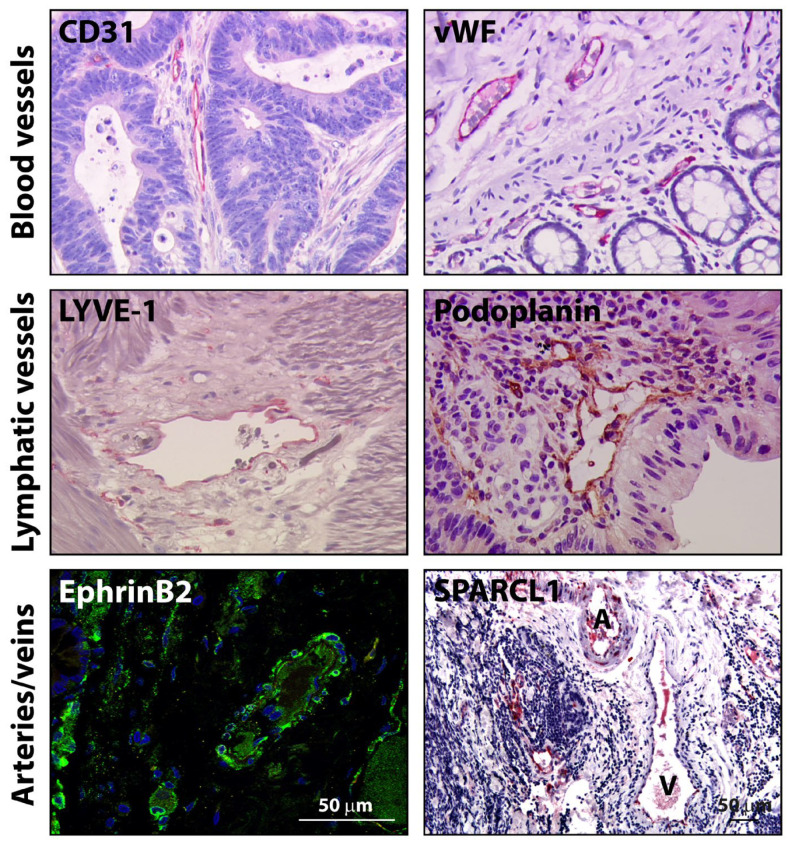
Different types of vessels are present in human colorectal cancer tissues. In human CRC, blood vessels can be labeled using the markers CD31 or vWF. Lymphatic vessels may be stained with LYVE-1 or podoplanin. Arteries and veins can be differentiated by labeling using the artery marker ephrinB2. This may be complemented by morphological differentiation of arteries (A) and veins (V) together with analysis of milieu-dependent expression of vessel markers such as SPARCL1. CD31, vWF, LYVE-1 and podoplanin panels: 25x objective; ephrinB2 and SPARCL1 panels: scale bars corresponding to 50 µm. The vWF panel is modified from Schellerer et al., Lab Invest 2007 [97].

Initially, compared with tumor cells, tumor endothelial cells were believed to be a superior therapeutic target because they are a more uniform, genetically stable and homogenous cell population. However, there is reasonable heterogeneity of endothelial cells in human tumor patients. First, tumor vessels differ from normal vessels, and within a single tumor, different types of vessels can be detected, such as blood and lymphatic vessels, arteries, veins and capillaries, together with vessels that regulate their function and protein expression in a milieu-dependent manner (Figure 2). Moreover, vessels may harbor different states of maturation and angiogenic activation and are composed of different types of ECs within a single vessel. For example, the hierarchical organization of angiogenically active vessels in tip and stalk cells with different phenotypes and functions is well accepted [89]. Therefore, it is obvious that specific targeting of the TEC population may be difficult. As an example of the therapeutic consequences of EC heterogeneity, it can be noted that the normal vasculature regresses along with the tumor vasculature upon treatment, indicating that not even TECs as a whole can be specifically targeted, not considering that different TEC populations exist [90]. Another example is vessel-associated pericytes/mural cells that contribute to vascular maturation and may protect vessels from antiangiogenic treatment, thereby fostering therapeutic resistance [77]. Moreover, compensatory functions of the lymphatic vessel system must be considered [91]. Notably, in preclinical studies, differentiating therapeutic responses with respect to different types of vessels or EC activation states has mostly not been considered. Disease- and patient-dependent heterogeneity of tumor endothelial cells has clearly been demonstrated by multiregion sequencing of renal carcinoma [92], and this approach may similarly apply to CRC. The abovementioned examples of EC heterogeneity, organ-dependent heterogeneity and the function of TECs are considered causes of differential responses to antiangiogenic treatment [93,94]. Finally, tumor vessel invasion may arise through different mechanisms, such as vessel co-option or vascular mimicry, which are not necessarily dependent on active angiogenesis and, accordingly, may result in resistance to antiangiogenic treatment approaches [95]. In conclusion, both TEC heterogeneity and the different mechanisms through which tumor cells arrange their supply of oxygen and nutrients cause the tumor vasculature to be a more complex target, as initially appreciated [77,78,96].

### 3.4. Tumor Microenvironment (TME)-Dependent Plasticity of ECs Involving Angiocrine Mediators

Moreover, in recent years, it has become clear that ECs in tumors are not just passive conduits of blood that deliver oxygen, nutrients and circulating cells or remove waste products from tissues. Endothelial cells are part of the tumor microenvironment (TME), which is defined by direct or indirect interactions through paracrine mediators of tumor cells with their surrounding stromal cells. These interactions can alter the phenotypes and functions of the involved cell populations, including TECs. It was shown that TECs in CRC are epigenetically imprinted in a TME-dependent manner, resulting in stably maintained differential phenotypes with an impact on patient prognosis [98].

Notably, the vasculature can release so-called “angiocrine” molecules that act on neighboring cells, including tumor cells, which may promote or counteract tumor growth. Tumor-supporting angiocrine activities were described for vascular-derived IL-6 in glioblastoma (GBM), which induces TME-dependent alternative macrophage polarization by activating HIF-2α and arginase-1, resulting in GBM progression [99], or for TEC-derived jagged-1, which induces B-cell lymphoma invasiveness and chemoresistance [79]. Speci-fically, the antitumorigenic functions of angiocrine tumor vessel activities may have an unappreciated clinical impact on the outcome of antiangiogenic therapy [100]. Thrombo-spondin-1 is an angiocrine molecule with antitumorigenic functions that has been shown to induce tumor dormancy in the perivascular niche in breast cancer [101]. Similarly, in CRC, the TEC-derived matricellular protein SPARCL1 is associated with a positive prognosis in patients and is suspected to mediate the antitumorigenic functions of tumor vessels [99,102]. Notably, two other angiocrine mediators, ANG2 and BMP2, have recently gained attention as novel targets because of the consideration of ANG2 inhibitors and Tie2 activators in the ANG/Tie pathway as drug candidates [103] or BMP2 as a target in the calcineurin/NFAT-axis [104]. These initial observations indicate that under certain conditions, not only inhibiting but also supporting tumor vessels may be advantageous for patients. Whether angiocrine antitumorigenic functions of tumor vessels also contribute to the positive effects of vessel normalization strategies warrants further investigation [105].

### 3.5. Induction of EC Anergy

In recent years, modulation of the immune response by induction of endothelial cell anergy has attracted increased amounts of attention. This lack of responsiveness to inflammatory signals termed “endothelial cell anergy” is associated with immune cell exclusion and the downregulation of adhesion molecules such as ICAM-1/VCAM-1, which enable tissue extravasation of immune cells [106]. The presence of EC anergy renders tumors less responsive to immunotherapy given in combination with antiangiogenic treatment. An immunosuppressive function of the endothelial barrier has been reported for galectin-1-driven T-cell exclusion in the tumor endothelium, promoting immunotherapy resistance [107]. Another molecule involved in this context is the death mediator Fas L, which is specifically detected in the tumor vasculature and cooperatively induced by VEGF and PGE_2,_ resulting in a tumor endothelial death barrier that promotes immune tolerance associated with low CD8+ T-cell levels. Immune tolerance can be pharmacologically attenuated by VEGF and PGE_2_ inhibition [108]. In conclusion, the presence of EC anergy under certain conditions in human patients results in immune tolerance, and immune cell exclusion is an issue hampering the response to antiangiogenic therapy.

### 3.6. Genomic Instability of TECs

Several reports have challenged the initial assumption that TECs are genetically stable. For example, in B-cell lymphoma patients, tumor cell-specific genetic alterations have been detected in microvascular endothelial cells [109]. Furthermore, vascular mimicry is presently defined as the autonomous formation of tumor vessels through tumor cells without the presence of a tumor endothelium. Vascular mimicry may coexist with mosaic vessels where the tumor endothelium is still partially integrated into the vessel wall [110]. Both types of tumor vasculature formation have implications for therapy by fostering resistance, as observed for vessels assembled by melanoma and endothelial cells [111]. Moreover, tumor cell differentiation into “endothelial-like” cells may occur and are considered an additional mode of therapeutic resistance. This was highlighted for GBM patients, in which endothelial cell-like cells were found to transdifferentiate from GBM stem-like cells [112,113,114]. Furthermore, increased aneuploidy was detected in TECs in human renal carcinoma [115] and in mouse melanoma models with high and low metastatic potential [116]. The potential genomic instability of TECs themselves or vessel walls made of tumor cells could be an issue as an escape and resistance mechanism limiting antiangiogenic treatment. However, this potential mode of resistance may be less relevant for CRC specifically. This assumption is based on the findings of a recent study in which potential genetic alterations in TECs were analyzed via systematic omics analyses in human CRC patients. Compared with their normal colon endothelial counterparts, corresponding PBMCs or tumor cells were found to be genetically stable from TECs isolated from these patients [98]. Furthermore, genetic drift of tumor-specific alterations to endothelial cells could not be detected, but the high load of mutations in MSI-positive patients warrants further investigation [97].

### 3.7. Imbalance of Intracellular Signaling Molecules (ROS, Calcium)

For a summary on the role of intracellular signaling and regulation in endothelial cells via the classical VEGFR/VEGF, FGFR/FGF, Tie2/Ang2, Notch/DLL4/Jagged1 and EphB/Ephrin B axes, we refer to a comprehensive review published recently [51]. In addition to these mainstream factors, altered concentrations of calcium and reactive oxygen species (ROS) may contribute to therapy resistance and side effects. ROS and calcium are two closely interconnected signaling molecules in eukaryotic cells, and calcium is known to modulate ROS homeostasis [117]. In CRC liver metastasis of bevacizumab-resistant patients, increased matrix stiffness was detected, which resulted in lipid metabolic cross-talk between the tumor and stromal cells characterized by increased levels of ROS and free fatty acids and a higher fatty acid oxidation rate, all of which contributed to bevacizumab resistance [118]. A ROS imbalance in HUVECs after anti-VEGF treatment was also reported by others [119]. Anti-VEGF treatment may increase calcium and ROS levels in parallel with decreased ATP production and increased cell damage, as has been observed in renal cells [120]. The addition of the calcium channel blocker benidipine to antiangiogenic treatment reduced renal toxicity, a known side effect of bevacizumab treatment [120]. Accordingly, modulating intracellular ROS and calcium imbalances during antiangiogenic treatment may help to overcome therapeutic resistance or side effects in the future.

### 3.8. Inadequate Preclinical Models and/or Limited Analysis

Until recently, for colorectal cancer, no or only very limited in vivo animal models existed that exhibit spontaneous distant metastasis similar to that of human patients. Considering that metastasis is the major cause of death in CRC patients and that the vasculature plays a key role in regulating tumor cell dissemination, optimizing antiangiogenic therapy regimens in appropriate model systems with distant metastasis is key. Meanwhile, novel organoid-based in vivo animal models that recapitulate spontaneous distant metastasis similar to that observed in human patients have been established; therefore, these models have great potential for improving preclinical screening [121,122]. In the future, novel targets are expected to be identified using such advanced models, and novel drug candidates can be evaluated in the preclinical setting, including distant metastasis. Furthermore, in the preclinical animal models used to date, the different types or activation states of ECs present in tumors have mostly not been analyzed with respect to differential treatment responses. This is a substantial issue that hampers the understanding of a potential milieu-dependent EC response to treatment and should be addressed in the future.

## 4. Conclusions and Future Opportunities for Antiangiogenic Tumor Therapy in CRC

After the first euphoria and the subsequent challenges in translating experimental results into clinical application, angiogenic inhibitors are currently an inherent part of therapy not only for mCRC but also for multiple other benign and malignant diseases. Nonetheless, almost 20 years after the first approval of bevacizumab for mCRC treatment, the results of antiangiogenic therapy for mCRC have been inconsistent, the effects have been less than initially expected and only five drugs have been approved. These are, within the class of monoclonal antibodies, bevacizumab, ramucirumab and aflibercept and, within the class of tyrosine kinase inhibitors, regorafenib and the new, promising TKI fruquintinib.

The prevailing challenges, which are complex and not adequately addressed yet, include the design of the therapy schedule, heterogeneity and TME-dependent plasticity of endothelial cells, the induction of immunosuppressive EC anergy, a low number of drug targets, deregulated intracellular signal mediators, a lack of stratification and response biomarkers, and the continued use of limited preclinical animal models lacking sporadic distant metastasis, the latter representing the major issue during disease progression and management.

Opportunities to overcome these challenges in the future include the integration of novel combinations of antiangiogenic drugs with immunotherapy, such as combined PD-1/VEGF blockade; novel application modes, such as metronomic dosing; or optimized low-dose combinations. Moreover, novel targets originating mostly from the pool of angiocrine proteins, such as those targeting the Tie2/Ang2 or Notch1/DLL4 axes, are promising for improving the efficacy of antiangiogenic treatment; these drugs are either in development or are already in clinical trials. Moreover, other angiocrine modulators, such as thrombospondin or SPARCL1, could be used as future therapeutic targets or biomarkers and may warrant further investigation in preclinical studies. Drugs targeting intracellular signal mediators deregulated during antiangiogenic treatment, such as calcium in combination with VEGF blockade, may also help to overcome therapeutic resistance or reduce side effects. Notably, improved tailored pretherapeutic drug testing with advanced tools, such as organoid-based animal models able to spontaneously metastasize in the periphery or the use of patient-derived organoids to individualized therapy, may help to overcome current issues. Most importantly, it will be necessary to consider, analyze and differentiate the impact of EC heterogeneity and the milieu-dependent plasticity of TECs in response to antiangiogenic therapy in more detail. This has to be considered in improved preclinical models with spontaneous distant metastasis to ultimately translate this aspect successfully into later clinical application.

## Figures and Tables

**Table 1:** Pivotal phase III clinical trials of antiangiogenic agents in treatment of mCRC.

Clinical Trial	Treatment	Indication	mOS, Months (95%CI)	mPFS, Months (95% CI)	ORR, %	HR (OS) (95%CI)	Ref.
AVF2107g	Beva + IFL Beva + placebo	1st line	20.3 (n.r.) 15.6 (n.r.)	10.6 (n.r.) 6.2 (n.r.)	44.8 34.8	0.66 (n.r.), *p* < 0.001	[19]
ITACa	Beva + FOLFOX/FOLFIRI FOLFOX/FOLFIRI	1st line	20.8 (15.9–23.2) 21.3 (19.9–24.1)	9.6 (8.2–10.3) 8.4 (7.2–9.0)	50.6 50	1.13 (0.89–1.43), *p* = 0.304	[21]
ML18147	Beva+FOLFOX/FOLFIRI FOLFOX/FOLFIRI	2nd line	11.2 (10.4–12.2) 9.8 (8.9–10.7)	5.7 (5.2–6.2) 4.1 (3.7–4.4)	5 4	0.81 (0.69–0.94), *p* = 0.0062)	[22]
VELOUR	Aflibercept + FOLFIRI FOLFIRI	2nd line	13.5 (12.52–14.95) 12.6 (11.07–13.11)	6.9 (6.51–7.2) 4.67 (4.21–5.36)	19.8 11.1	0.817 (0.713–0.937), *p* = 0.0032)	[23]
RAISE	Ramucirumab + FOLFIRI FOLFIRI	2nd line	13.3 (12.4–14.5) 11.7 (10.8–12–7)	5.7 (5.5–6.2) 4.5 (4.2–5.4)	13.4 12.5	0.844 (0.73–0.976), *p* = 0.0219	[24]
CORRECT	Regorafenib Placebo	refractory	6.4 (CI n.r.) 5.0 (CI n.r.)	1.9 (CI n.r.) 1.7 (CI n.r.)	1 0.4	0.77 (0.64–0.94), *p* = 0.0052	[25]
FRESCO II	Fruquintinib Placebo	3rd/later line	7.4 (6.7–8.2) 4.8 (4.0–5.8)	3.7 (3.5–3.8) 1.8 (1.8–1.9)	5 0	0.66 (0.55–0.80), *p* < 0.0001	[26]

mOS: median overall survival. mPFS: median progression-free survival. ORR: objective response rate. HR (OS): hazard ratio of overall survival. CI: confidence interval. Beva: bevacizumab. FOLFOX: 5-fluorouracil, leucovorin, oxaliplatin. FOLFIRI: 5-fluorouracil, leucovorin, irinotecan. IFL: irinotecan, bolus fluorouracil, leucovorin. n.r.: not reported.

**Table 2:** Selection of antiangiogenic drugs in phase II/III trials in the last decade not receiving clinical approval.

Drug	Target	Regimen	Phase	Indication	Results in CRC	Ref.
**TKI**						
Brivanib	VEGFR-2, -3, FGFR-1, -2, -3	Brivanib/cetuximab Placebo/cetuximab	III	Refractory	No improvement of OS, significant improvement of ORR and PFS, increased toxicity	[55]
Cediranib	VEGFR-1, -2, -3, PDGFRβ, KIT	Cediranib/FOLFOX Beva/FOLFOX	II	2nd line	No improvement of PFS or OS	[56]
		Cediranib/FOLFOX or CAPOX Placebo/FOLFOX or CAPOX	III	1st line	Modest PFS prolongation, no impact on OS	[57]
		Cediranib/FOLFOX Beva/FOLFOX	II/III	1st line	PFS and OS comparable to those of beva, less favorable profile of adverse events	[58]
Linifanib	VEGFR-1, -2, -3, PDGFRβ	Linifanib/FOLFOX Beva/FOLFOX	II	2nd line	PFS and OS comparable to those of beva, more adverse events	[59]
Tivozanib	VEGFR-1, -2, -3, KIT, PDGFRβ	Tivozanib/FOLFOX Beva/FOLFOX	II	1st line	Efficacy comparable to that of beva	[60]
Vandetanib	EGFR, VEGFR-2, RET, BRK, TIE-2	Vandetanib/FOLFOX Placebo/FOLFOX	II	2nd line	No efficacy	[61]
Vatalanib	VEGFR-1, -2, -3	Vatalanib/FOLFOX Placebo/FOLFOX	III	1st line	No efficacy in OS, PFS, ORR	[62]
		Vatalanib/FOLFOX Placebo/FOLFOX	III	2nd line	Improvement of PFS, but not OS	[63]
Famitinib	VEGFR-2, -3, KIT, PDGFR, RET	Famitinib Placebo	II	3rd or later line	Prolongation of PFS, no improvement of OS	[64]
Nintedanib	VEGFR-1, -2, -3, FGFR-1, -2, -3, PDGFRα/β	Nintedanib/FOLFOX Beva/FOLFOX	I/II	1st line	Similar PFS	[65]
		Nintedanib/FOLFOX Placebo/FOLFOX	II	2nd line	Nonsignificant trend for improved PFS, OS, DCR	[66]
		Nintedanib Placebo	III	Refractory	No improvement of OS, modest increase of PFS	[67]
**Monoclonal antibodies**						
Axitinib	VEGFR-1, -2, -3	Axitinib/FOLFOX Beva/FOLFO Axitinib/Beva/FOLFOX	II	1st line	No improvement of ORR, PFS or OS by addition of axitinib or combination with beva	[68]
		Axitinib vs. placebo	II	Maintenance	Significantly longer PFS with axitinib	[69]
		Axitinib/FOLFOX Beva/FOLFOX Axitinib/FOLFIRI Beva/FOLFIRI	II	2nd line	No improvement of PFS and OR, but more adverse events with axitinib	[70]
Parsatuzumab	EGFL7	Parsatuzumab/FOLFOX/beva Placebo/FOLFOX/beva	II	1st line	No improvement of ORR, PFS, OS	[71]
Vanucizumab	VEGF-A, Ang-2	Vanucizumab/FOLFOX Placebo/FOLFOX	II	1st line	No improvement of PFS, increased toxicity	[72]
**Peptibody**						
Trebananib	Ang-1, -2	Trebananib/FOLFIRI Placebo/FOLFIRI	II	2nd line	No improvement of OS or PFS	[73]

CRC: colorectal cancer. VEGFR: vascular endothelial growth factor receptor. FGFR: fibroblast growth factor receptor. FOLFOX: 5-fluorouracil, leucovorin, oxaliplatin. FOLFIRI: 5-fluorouracil, leucovorin, irinotecan. OS: overall survival. ORR: objective response rate. PDGFRβ: platelet-derived growth factor β. Beva: bevacizumab. CAPOX: capecitabine, oxaliplatin. PFS: progression-free survival. KIT: tyrosine protein kinase KIT. EGFR: epidermal growth factor receptor. RET: rearranged during transfection. BRK: breast tumor kinase. TIE-2: EGFL7: Ang: angiopoietin.

## References

[1] World Health Organization (2023). Colorectal Cancer. https://www.who.int/news-room/fact-sheets/detail/colorectal-cancer.

[2] Arnold M., Abnet C.C., Neale R.E., Vignat J., Giovannucci E.L., McGlynn K.A., Bray F. (2020). Global Burden of 5 Major Types of Gastrointestinal Cancer. Gastroenterology.

[3] Ugai T., Sasamoto N., Lee H.-Y., Ando M., Song M., Tamimi R.M., Kawachi I., Campbell P.T., Giovannucci E.L., Weiderpass E. (2022). Is early-onset cancer an emerging global epidemic? Current evidence and future implications. Nat. Rev. Clin. Oncol..

[4] Cervantes A., Adam R., Roselló S., Arnold D., Normanno N., Taïeb J., Seligmann J., De Baere T., Osterlund P., Yoshino T. (2022). Metastatic colorectal cancer: ESMO Clinical Practice Guideline for di-agnosis, treatment and follow-up. Ann. Oncol..

[5] Hanahan D., Weinberg R.A. (2011). Hallmarks of cancer: The next generation. Cell.

[6] Folkman J. (1971). Tumor angiogenesis: Therapeutic implications. N. Engl. J. Med..

[7] Folkman J., Vincent S.H., De Vita T., Rosenberg S.A. (2001). Antiangiogenesis Agents, in Cancer: Principles and Practice of Oncology.

[8] Cao Y., Langer R. (2008). A review of Judah Folkman’s remarkable achievements in biomedicine. Proc. Natl. Acad. Sci. USA.

[9] Ribatti D. (2008). Judah Folkman, a pioneer in the study of angiogenesis. Angiogenesis.

[10] Hanson F.R., Eble T.E. (1949). An Antiphage Agent Isolated from *Aspergillus* sp. J. Bacteriol..

[11] Guruceaga X., Perez-Cuesta U., Cerio A.D.D., Gonzalez O., Rementeria A. (2019). Fumagillin, a Mycotoxin of Aspergillus fumigatus: Biosynthesis, Biological Activities, Detection, and Ap-plications. Toxins.

[12] Ingber D., Fujita T., Kishimoto S., Sudo K., Kanamaru T., Brem H., Folkman J. (1990). Synthetic analogues of fumagillin that inhibit angiogenesis and suppress tumour growth. Nature.

[13] O’Reilly M.S., Boehm T., Shing Y., Fukai N., Vasios G., Lane W.S., Flynn E., Birkhead J.R., Olsen B.R., Folkman J. (1997). Endostatin: An Endogenous Inhibitor of Angiogenesis and Tumor Growth. Cell.

[14] O’Reilly M.S., Holmgren L., Shing Y., Chen C., Rosenthal R.A., Moses M., Lane W.S., Cao Y., Sage E., Folkman J. (1994). Angiostatin: A novel angiogenesis inhibitor that mediates the suppression of metastases by a lewis lung carcinoma. Cell.

[15] O’Reilly M.S., Holmgren L., Chen C., Folkman J. (1996). Angiostatin induces and sustains dormancy of human primary tumors in mice. Nat. Med..

[16] Boehm T., Folkman J., Browder T., O’Reilly M.S. (1997). Antiangiogenic therapy of experimental cancer does not induce acquired drug resistance. Nature.

[17] Kolata G. (1998). Hope in the Lab: A Special Report; A Cautious Awe Greets Drugs That Eradicate Tumors in Mice. The New York Times.

[18] Watson J.D. (1998). Opinion, High Hopes on Cancer, Letter to the Editor. The New York Times.

[19] Hurwitz H., Fehrenbacher L., Novotny W., Cartwright T., Hainsworth J., Heim W., Berlin J., Baron A., Griffing S., Holmgren E. (2004). Bevacizumab plus Irinotecan, Fluorouracil, and Leucovorin for Metastatic Colorectal Cancer. N. Engl. J. Med..

[20] Hansen T.F., Qvortrup C., Pfeiffer P. (2021). Angiogenesis Inhibitors for Colorectal Cancer. A Review of the Clinical Data. Cancers.

[21] Passardi A., Nanni O., Tassinari D., Turci D., Cavanna L., Fontana A., Ruscelli S., Mucciarini C., Lorusso V., Ragazzini A. (2015). Effectiveness of bevacizumab added to standard chemotherapy in metastatic colorectal cancer: Final results for first-line treatment from the ITACa randomized clinical trial. Ann. Oncol..

[22] Bennouna J., Sastre J., Arnold D., Österlund P., Greil R., Van Cutsem E., von Moos R., Viéitez J.M., Bouché O., Borg C. (2013). Continuation of bevacizumab after first progression in metastatic colorectal cancer (ML18147): A randomised phase 3 trial. Lancet Oncol..

[23] Van Cutsem E., Tabernero J., Lakomy R., Prenen H., Prausová J., Macarulla T., Ruff P., van Hazel G.A., Moiseyenko V., Ferry D. (2012). Addition of Aflibercept to Fluorouracil, Leucovorin, and Irinotecan Improves Survival in a Phase III Randomized Trial in Patients with Metastatic Colorectal Cancer Previously Treated With an Oxaliplatin-Based Regimen. J. Clin. Oncol..

[24] Tabernero J., Yoshino T., Cohn A.L., Obermannova R., Bodoky G., Garcia-Carbonero R., Ciuleanu T.E., Portnoy D.C., Cutsem E.V., Grothey A. (2015). Ramucirumab versus placebo in combination with second-line FOLFIRI in patients with metastatic colo-rectal carcinoma that progressed during or after first-line therapy with bevacizumab, oxaliplatin, and a fluoropyrimidine (RAISE): A randomised, double-blind, multicentre, phase 3 study. Lancet Oncol..

[25] Grothey A., Van Cutsem E., Sobrero A., Siena S., Falcone A., Ychou M., Humblet Y., Bouché O., Mineur L., Barone C. (2013). Regorafenib monotherapy for previously treated metastatic colorectal cancer (CORRECT): An international, multicentre, randomised, placebo-controlled, phase 3 trial. Lancet.

[26] Dasari A., Lonardi S., Garcia-Carbonero R., Elez E., Yoshino T., Sobrero A., Yao J., García-Alfonso P., Kocsis J., Gracian A.C. (2023). Fruquintinib versus placebo in patients with refractory metastatic colorectal cancer (FRESCO-2): An interna-tional, multicentre, randomised, double-blind, phase 3 study. Lancet.

[27] Garcia J., Hurwitz H.I., Sandler A.B., Miles D., Coleman R.L., Deurloo R., Chinot O.L. (2020). Bevacizumab (Avastin®) in cancer treatment: A review of 15 years of clinical experience and future outlook. Cancer Treat. Rev..

[28] Watanabe T., Itabashi M., Shimada Y., Tanaka S., Ito Y., Ajioka Y., Hamaguchi T., Hyodo I., Igarashi M., Ishida H. (2020). Japanese Society for Cancer of the Colon and Rectum (JSCCR) guidelines 2019 for the treatment of colorectal cancer. Int. J. Clin. Oncol..

[29] Benson A.B., Venook A.P., Al-Hawary M.M., Arain M.A., Chen Y.J., Ciombor K.K., Cohen S., Cooper H.S., Deming D., Farkas L. (2021). Colon Cancer, Version 2.2021, NCCN Clinical Practice Guidelines in Oncology. J. Natl. Compr. Cancer Netw..

[30] Chen H.X., Cleck J.N. (2009). Adverse effects of anticancer agents that target the VEGF pathway. Nat. Rev. Clin. Oncol..

[31] Souglakos J., Ziras N., Kakolyris S., Boukovinas I., Kentepozidis N., Makrantonakis P., Xynogalos S., Christophyllakis C., Kouroussis C., Vamvakas L. (2012). Randomised phase-II trial of CAPIRI (capecitabine, irinotecan) plus bevacizumab vs FOLFIRI (folinic acid, 5-fluorouracil, irinotecan) plus bevacizumab as first-line treatment of patients with unresectable/metastatic colorectal cancer (mCRC). Br. J. Cancer.

[32] Van Cutsem E., Rivera F., Berry S., Kretzschmar A., Michael M., DiBartolomeo M., Mazier M.-A., Canon J.-L., Georgoulias V., Peeters M. (2009). Safety and efficacy of first-line bevacizumab with FOLFOX, XELOX, FOLFIRI and fluoropyrimidines in metastatic colorectal cancer: The BEAT study. Ann. Oncol..

[33] Tampellini M., Sonetto C., Scagliotti G.V. (2016). Novel anti-angiogenic therapeutic strategies in colorectal cancer. Expert Opin. Investig. Drugs.

[34] Kabbinavar F. (2003). Phase II, randomized trial comparing bevacizumab plus fluorouracil (FU)/leucovorin (LV) with FU/LV alone in patients with metastatic colorectal cancer. J. Clin. Oncol..

[35] Cunningham D., Lang I., Marcuello E., Lorusso V., Ocvirk J., Shin D.B., Jonker D., Osborne S., Andre N., Waterkamp D. (2013). Bevacizumab plus capecitabine versus capecitabine alone in elderly patients with previously untreated metastatic colorectal cancer (AVEX): An open-label, randomised phase 3 trial. Lancet Oncol..

[36] Kabbinavar F.F., Schulz J., McCleod M., Patel T., Hamm J.T., Hecht J.R., Mass R., Perrou B., Nelson B., Novotny W.F. (2005). Addition of Bevacizumab to Bolus Fluorouracil and Leucovorin in First-Line Metastatic Colorectal Cancer: Results of a Randomized Phase II Trial. J. Clin. Oncol..

[37] Saltz L.B., Clarke S., Diaz-Rubio E., Scheithauer W., Figer A., Wong R., Koski S., Lichinitser M., Yang T.-S., Rivera F. (2008). Bevacizumab in Combination With Oxaliplatin-Based Chemotherapy As First-Line Therapy in Metastatic Colorectal Cancer: A Randomized Phase III Study. J. Clin. Oncol..

[38] Tebbutt N.C., Wilson K., Gebski V.J., Cummins M.M., Zannino D., van Hazel G.A., Robinson B., Broad A., Ganju V., Ackland S.P. (2010). Capecitabine, Bevacizumab, and Mitomycin in First-Line Treatment of Metastatic Colorectal Cancer: Results of the Australasian Gastrointestinal Trials Group Randomized Phase III MAX Study. J. Clin. Oncol..

[39] Baraniskin A., Buchberger B., Pox C., Graeven U., Holch J.W., Schmiegel W., Heinemann V. (2019). Efficacy of bevacizumab in first-line treatment of metastatic colorectal cancer: A systematic review and meta-analysis. Eur. J. Cancer.

[40] Heinemann V., von Weikersthal L.F., Decker T., Kiani A., Vehling-Kaiser U., Al-Batran S.E., Heintges T., Lerchenmüller C., Kahl C., Seipelt G. (2014). FOLFIRI plus cetuximab versus FOLFIRI plus bevacizumab as first-line treatment for patients with metastatic colorectal cancer (FIRE-3): A randomised, open-label, phase 3 trial. Lancet Oncol..

[41] Venook A.P., Niedzwiecki D., Lenz H.J., Innocenti F., Fruth B., Meyerhardt J.A., Schrag D., Greene C., O’Neil B.H., Atkins J.N. (2017). Effect of First-Line Chemotherapy Combined with Cetuximab or Bevacizumab on Overall Survival in Patients with KRAS Wild-Type Advanced or Metastatic Colorectal Cancer: A Randomized Clinical Trial. JAMA.

[42] Schwartzberg L.S., Rivera F., Karthaus M., Fasola G., Canon J.-L., Hecht J.R., Yu H., Oliner K.S., Go W.Y. (2014). PEAK: A Randomized, Multicenter Phase II Study of Panitumumab Plus Modified Fluorouracil, Leucovorin, and Oxaliplatin (mFOLFOX6) or Bevacizumab Plus mFOLFOX6 in Patients with Previously Untreated, Unresectable, Wild-Type *KRAS* Exon 2 Metastatic Colorectal Cancer. J. Clin. Oncol..

[43] Holch J.W., Ricard I., Stintzing S., Modest D.P., Heinemann V. (2016). The relevance of primary tumour location in patients with metastatic colorectal cancer: A meta-analysis of first-line clinical trials. Eur. J. Cancer.

[44] Loupakis F., Hurwitz H.I., Saltz L., Arnold D., Grothey A., Nguyen Q.L., Osborne S., Talbot J., Srock S., Lenz H.-J. (2018). Impact of primary tumour location on efficacy of bevacizumab plus chemotherapy in metastatic colorectal cancer. Br. J. Cancer.

[45] Hegewisch-Becker S., Graeven U., A Lerchenmüller C., Killing B., Depenbusch R., Steffens C.-C., Al-Batran S.-E., Lange T., Dietrich G., Stoehlmacher J. (2015). Maintenance strategies after first-line oxaliplatin plus fluoropyrimidine plus bevacizumab for patients with metastatic colorectal cancer (AIO 0207): A randomised, non-inferiority, open-label, phase 3 trial. Lancet Oncol..

[46] Masi G., Salvatore L., Boni L., Loupakis F., Cremolini C., Fornaro L., Schirripa M., Cupini S., Barbara C., Safina V. (2015). Continuation or reintroduction of bevacizumab beyond progression to first-line therapy in metastatic colorectal cancer: Final results of the randomized BEBYP trial. Ann. Oncol..

[47] Prager G.W., Taieb J., Fakih M., Ciardiello F., Van Cutsem E., Elez E., Cruz F.M., Wyrwicz L., Stroyakovskiy D., Pápai Z. (2023). Trifluridine–Tipiracil and Bevacizumab in Refractory Metastatic Colorectal Cancer. N. Engl. J. Med..

[48] Otsu S., Hironaka S. (2023). Current Status of Angiogenesis Inhibitors as Second-Line Treatment for Unresectable Colorectal Cancer. Cancers.

[49] Hashimoto T., Otsu S., Hironaka S., Takashima A., Mizusawa J., Kataoka T., Fukuda H., Tsukamoto S., Hamaguchi T., Kanemitsu Y. (2023). Phase II biomarker identification study of anti-VEGF agents with FOLFIRI for pretreated metastatic colorectal cancer. Future Oncol..

[50] Kumar R., Crouthamel M.-C., Rominger D.H., Gontarek R.R., Tummino P.J., A Levin R., King A.G. (2009). Myelosuppression and kinase selectivity of multikinase angiogenesis inhibitors. Br. J. Cancer.

[51] Cao Y., Langer R., Ferrara N. (2023). Targeting angiogenesis in oncology, ophthalmology and beyond. Nat. Rev. Drug Discov..

[52] Zhang Y., Zou J.Y., Wang Z., Wang Y. (2019). Fruquintinib: A novel antivascular endothelial growth factor receptor tyrosine kinase inhibitor for the treatment of metastatic colorectal cancer. Cancer Manag. Res..

[53] Li J., Qin S., Xu R.H., Shen L., Xu J., Bai Y., Yang L., Deng Y., Chen Z.D., Zhong H. (2018). Effect of Fruquintinib vs Placebo on Overall Survival in Patients with Previously Treated Metastatic Colorectal Cancer: The FRESCO Randomized Clinical Trial. JAMA.

[54] Shirley M. (2018). Fruquintinib: First Global Approval. Drugs.

[55] Siu L.L., Shapiro J.D., Jonker D.J., Karapetis C.S., Zalcberg J.R., Simes J., Couture F., Moore M.J., Price T.J., Siddiqui J. (2013). Phase III Randomized, Placebo-Controlled Study of Cetuximab Plus Brivanib Alaninate Versus Cetuximab Plus Placebo in Patients With Metastatic, Chemotherapy-Refractory, Wild-Type *K-RAS* Colorectal Carcinoma: The NCIC Clinical Trials Group and AGITG CO.20 Trial. J. Clin. Oncol..

[56] Cunningham D., Wong R.P., D’Haens G., Douillard J.Y., Robertson J., Stone A.M., Van Cutsem E. (2013). Cediranib with mFOLFOX6 vs bevacizumab with mFOLFOX6 in previously treated metastatic colo-rectal cancer. Br. J. Cancer.

[57] Hoff P.M., Hochhaus A., Pestalozzi B.C., Tebbutt N.C., Li J., Kim T.W., Koynov K.D., Kurteva G., Pintér T., Cheng Y. (2012). Cediranib Plus FOLFOX/CAPOX Versus Placebo Plus FOLFOX/CAPOX in Patients with Previously Untreated Metastatic Colorectal Cancer: A Randomized, Double-Blind, Phase III Study (HORIZON II). J. Clin. Oncol..

[58] Schmoll H.-J., Cunningham D., Sobrero A., Karapetis C.S., Rougier P., Koski S.L., Kocakova I., Bondarenko I., Bodoky G., Mainwaring P. (2012). Cediranib With mFOLFOX6 Versus Bevacizumab With mFOLFOX6 As First-Line Treatment for Patients with Advanced Colorectal Cancer: A Double-Blind, Randomized Phase III Study (HORIZON III). J. Clin. Oncol..

[59] O’Neil B.H., Cainap C., Van Cutsem E., Gorbunova V., Karapetis C.S., Berlin J., Goldberg R.M., Qin Q., Qian J., Ricker J.L. (2014). Randomized Phase II Open-Label Study of mFOLFOX6 in Combination with Linifanib or Bevacizumab for Metastatic Colorectal Cancer. Clin. Color. Cancer.

[60] Benson A.B., Kiss I., Bridgewater J., Eskens F.A., Sasse C., Vossen S., Chen J., Van Sant C., Ball H.A., Keating A. (2016). BATON-CRC: A Phase II Randomized Trial Comparing Tivozanib Plus mFOLFOX6 with Bevacizumab Plus mFOLFOX6 in Stage IV Metastatic Colorectal Cancer. Clin. Cancer Res..

[61] Yang T.S., Oh D.Y., Guimbaud R., Szanto J., Salek T., Thurzo L., Vieitez J.M., Pover G.M., Kim T.W. (2009). Vandetanib plus mFOLFOX6 in patients with advanced colorectal cancer (CRC): A randomized, double-blind, placebo-controlled phase II study. J. Clin. Oncol..

[62] Hecht J.R., Trarbach T., Hainsworth J.D., Major P., Jäger E., Wolff R.A., Lloyd-Salvant K., Bodoky G., Pendergrass K., Berg W. (2011). Randomized, placebo-controlled, phase III study of first-line oxaliplatin-based chemotherapy plus PTK787/ZK 222584, an oral vascular endothelial growth factor receptor inhibitor, in patients with metastatic colorectal adenocarcinoma. J. Clin. Oncol..

[63] Van Cutsem E., Bajetta E., Valle J., Köhne C.H., Hecht J.R., Moore M., Germond C., Berg W., Chen B.L., Jalava T. (2011). Randomized, placebo-controlled, phase III study of oxaliplatin, fluorouracil, and leucovorin with or without PTK787/ZK 222584 in patients with previously treated metastatic colorectal adenocarcinoma. J. Clin. Oncol..

[64] Xu R.-H., Shen L., Wang K.-M., Wu G., Shi C.-M., Ding K.-F., Lin L.-Z., Wang J.-W., Xiong J.-P., Wu C.-P. (2017). Famitinib versus placebo in the treatment of refractory metastatic colorectal cancer: A multicenter, randomized, double-blinded, placebo-controlled, phase II clinical trial. Chin. J. Cancer.

[65] Van Cutsem E., Prenen H., D’Haens G., Bennouna J., Carrato A., Ducreux M., Bouché O., Sobrero A., Latini L., Staines H. (2015). A phase I/II, open-label, randomised study of nintedanib plus mFOLFOX6 versus bevacizumab plus mFOLFOX6 in first-line metastatic colorectal cancer patients. Ann. Oncol..

[66] Ettrich T.J., Perkhofer L., Decker T., Hofheinz R.D., Heinemann V., Hoffmann T., Hebart H.F., Herrmann T., Hannig C.V., Büchner-Steudel P. (2021). Nintedanib plus mFOLFOX6 as second-line treatment of metastatic, chemorefractory colorectal cancer: The randomised, placebo-controlled, phase II TRICC-C study (AIO-KRK-0111). Int. J. Cancer.

[67] Van Cutsem E., Yoshino T., Lenz H., Lonardi S., Falcone A., Limón M., Saunders M., Sobrero A., Park Y., Ferreiro R. (2018). Nintedanib for the treatment of patients with refractory metastatic colorectal cancer (LUME-Colon 1): A phase III, international, randomized, placebo-controlled study. Ann. Oncol..

[68] Infante J.R., Reid T.R., Cohn A.L., Edenfield W.J., Cescon T.P., Hamm J.T., Malik I.A., Rado T.A., McGee P.J., Richards D.A. (2013). Axitinib and/or bevacizumab with modified FOLFOX-6 as first-line therapy for metastatic colorectal cancer: A randomized phase 2 study. Cancer.

[69] Grávalos C., Carrato A., Tobeña M., Rodriguez-Garrote M., Soler G., Vieitez J.M., Robles L., Valladares-Ayerbes M., Polo E., Limón M.L. (2018). A Randomized Phase II Study of Axitinib as Maintenance Therapy After First-line Treatment for Metastatic Colorectal Cancer. Clin. Color. Cancer.

[70] Bendell J.C., Tournigand C., Swieboda-Sadlej A., Barone C., Wainberg Z.A., Kim J.G., Pericay C., Pastorelli D., Tarazi J., Rosbrook B. (2013). Axitinib or Bevacizumab Plus FOLFIRI or Modified FOLFOX-6 After Failure of First-Line Therapy for Metastatic Colorectal Cancer: A Randomized Phase II Study. Clin. Color. Cancer.

[71] García-Carbonero R., van Cutsem E., Rivera F., Jassem J., Gore I., Tebbutt N., Braiteh F., Argiles G., Wainberg Z., Funke R. (2017). Randomized Phase II Trial of Parsatuzumab (Anti-EGFL7) or Placebo in Combination with FOLFOX and Bevacizumab for First-Line Metastatic Colorectal Cancer. Oncologist.

[72] Bendell J.C., Sauri T., Gracián A.C., Alvarez R., Lopez C.L., García-Alfonso P., Hussein M., Miron M.L., Cervantes A., Montagut C. (2019). The McCAVE Trial: Vanucizumab plus mFOLFOX-6 Versus Bevacizumab plus mFOLFOX-6 in Patients with Previously Untreated Metastatic Colorectal Carcinoma (mCRC). Oncologist.

[73] Peeters M., Strickland A.H., Lichinitser M., Suresh A.V.S., Manikhas G., Shapiro J., Rogowski W., Huang X., Wu B., Warner D. (2013). A randomised, double-blind, placebo-controlled phase 2 study of trebananib (AMG 386) in combination with FOLFIRI in patients with previously treated metastatic colorectal carcinoma. Br. J. Cancer.

[74] Martin J.D., Seano G., Jain R.K. (2019). Normalizing Function of Tumor Vessels: Progress, Opportunities, and Challenges. Annu. Rev. Physiol..

[75] Jain R.K. (2005). Normalization of Tumor Vasculature: An Emerging Concept in Antiangiogenic Therapy. Science.

[76] Winkler F., Kozin S.V., Tong R.T., Chae S.-S., Booth M.F., Garkavtsev I., Xu L., Hicklin D.J., Fukumura D., di Tomaso E. (2004). Kinetics of vascular normalization by VEGFR2 blockade governs brain tumor response to radiation: Role of oxygenation, angiopoietin-1, and matrix metalloproteinases. Cancer Cell.

[77] Helfrich I., Scheffrahn I., Bartling S., Weis J., von Felbert V., Middleton M., Kato M., Ergün S., Augustin H.G., Schadendorf D. (2010). Resistance to antiangiogenic therapy is directed by vascular phenotype, vessel stabilization, and maturation in malignant melanoma. J. Exp. Med..

[78] Frentzas S., Simoneau E., Bridgeman V.L., Vermeulen P.B., Foo S., Kostaras E., Nathan M.R., Wotherspoon A., Gao Z.-H., Shi Y. (2016). Vessel co-option mediates resistance to anti-angiogenic therapy in liver metastases. Nat. Med..

[79] Cao Z., Ding B.-S., Guo P., Lee S.B., Butler J.M., Casey S.C., Simons M., Tam W., Felsher D.W., Shido K. (2014). Angiocrine Factors Deployed by Tumor Vascular Niche Induce B Cell Lymphoma Invasiveness and Chemoresistance. Cancer Cell.

[80] Kerbel R.S., Shaked Y. (2017). The potential clinical promise of ‘multimodality’ metronomic chemotherapy revealed by preclinical studies of metastatic disease. Cancer Lett..

[81] Cremolini C., Marmorino F., Bergamo F., Aprile G., Salvatore L., Masi G., Dell’aquila E., Antoniotti C., Murgioni S., Allegrini G. (2019). Phase II randomised study of maintenance treatment with bevacizumab or bevacizumab plus metronomic chemotherapy after first-line induction with FOLFOXIRI plus Bevacizumab for metastatic colorectal cancer patients: The MOMA trial. Eur. J. Cancer.

[82] Li Q., Wang Y., Jia W., Deng H., Li G., Deng W., Chen J., Kim B.Y., Jiang W., Liu Q. (2020). Low-Dose Anti-Angiogenic Therapy Sensitizes Breast Cancer to PD-1 Blockade. Clin. Cancer Res..

[83] Mettu N.B., Ou F.S., Zemla T.J., Halfdanarson T.R., Lenz H.J., Breakstone R.A., Boland P.M., Crysler O.V., Wu C., Nixon A.B. (2022). Assessment of Capecitabine and Bevacizumab with or Without Atezolizumab for the Treatment of Refractory Metastatic Colorectal Cancer: A Randomized Clinical Trial. JAMA Netw. Open.

[84] Lamplugh Z., Fan Y. (2021). Vascular Microenvironment, Tumor Immunity and Immunotherapy. Front. Immunol..

[85] Schmittnaegel M., Rigamonti N., Kadioglu E., Cassará A., Rmili C.W., Kiialainen A., Kienast Y., Mueller H.-J., Ooi C.-H., Laoui D. (2017). Dual angiopoietin-2 and VEGFA inhibition elicits antitumor immunity that is enhanced by PD-1 checkpoint blockade. Sci. Transl. Med..

[86] Di Tacchio M., Macas J., Weissenberger J., Sommer K., Bahr O., Steinbach J.P., Senft C., Seifert V., Glas M., Herrlinger U. (2019). Tumor Vessel Normalization, Immunostimulatory Reprogramming, and Improved Survival in Glio-blastoma with Combined Inhibition of PD-1, Angiopoietin-2, and VEGF. Cancer Immunol. Res..

[87] Shigeta K., Datta M., Hato T., Kitahara S., Chen I.X., Matsui A., Kikuchi H., Mamessier E., Aoki S., Ramjiawan R.R. (2019). Dual Programmed Death Receptor-1 and Vascular Endothelial Growth Factor Receptor-2 Blockade Promotes Vascular Normalization and Enhances Antitumor Immune Responses in Hepatocellular Carcinoma. Hepatology.

[88] Weiss A., Ding X., van Beijnum J.R., Wong I., Wong T.J., Berndsen R.H., Dormond O., Dallinga M., Shen L., Schlingemann R.O. (2015). Rapid optimization of drug combinations for the optimal angiostatic treatment of cancer. Angiogenesis.

[89] Gerhardt H., Golding M., Fruttiger M., Ruhrberg C., Lundkvist A., Abramsson A., Jeltsch M., Mitchell C., Alitalo K., Shima D. (2003). VEGF guides angiogenic sprouting utilizing endothelial tip cell filopodia. J. Cell Biol..

[90] Yang Y., Zhang Y., Cao Z., Ji H., Yang X., Iwamoto H., Wahlberg E., Länne T., Sun B., Cao Y. (2013). Anti-VEGF– and anti-VEGF receptor–induced vascular alteration in mouse healthy tissues. Proc. Natl. Acad. Sci. USA.

[91] Huang X., Bai X., Cao Y., Wu J., Huang M., Tang D., Tao S., Zhu T., Liu Y., Yang Y. (2010). Lymphoma endothelium preferentially expresses Tim-3 and facilitates the progression of lymphoma by me-diating immune evasion. J. Exp. Med..

[92] Gerlinger M., Rowan A.J., Horswell S., Math M., Larkin J., Endesfelder D., Gronroos E., Martinez P., Matthews N., Stewart A. (2012). Intratumor heterogeneity and branched evolution revealed by multiregion sequencing. N. Engl. J. Med..

[93] Nolan D.J., Ginsberg M., Israely E., Palikuqi B., Poulos M.G., James D., Ding B.-S., Schachterle W., Liu Y., Rosenwaks Z. (2013). Molecular Signatures of Tissue-Specific Microvascular Endothelial Cell Heterogeneity in Organ Maintenance and Regeneration. Dev. Cell.

[94] Zhao Q., Eichten A., Parveen A., Adler C., Huang Y., Wang W., Ding Y., Adler A., Nevins T., Ni M. (2018). Single-Cell Transcriptome Analyses Reveal Endothelial Cell Heterogeneity in Tumors and Changes following Antiangiogenic Treatment. Cancer Res..

[95] Van Beijnum J.R., Nowak-Sliwinska P., Huijbers E.J., Thijssen V.L., Griffioen A.W. (2015). The great escape; the hallmarks of resistance to antiangiogenic therapy. Pharmacol. Rev..

[96] Bridgeman V.L., Vermeulen P.B., Foo S., Bilecz A., Daley F., Kostaras E., Nathan M.R., Wan E., Frentzas S., Schweiger T. (2016). Vessel co-option is common in human lung metastases and mediates resistance to anti-angiogenic therapy in preclinical lung metastasis models. J. Pathol..

[97] Schellerer V.S., Croner R.S., Weinländer K., Hohenberger W., Stürzl M., Naschberger E. (2007). Endothelial cells of human colorectal cancer and healthy colon reveal phenotypic differences in culture. Lab Investig..

[98] Naschberger E., Fuchs M., Dickel N., Kunz M., Popp B., Anchang C.G., Demmler R., Lyu Y., Uebe S., Ekici A.B. (2023). Tumor microenvironment-dependent epigenetic imprinting in the vasculature predicts colon cancer outcome. Cancer Commun..

[99] Wang Q., He Z., Huang M., Liu T., Wang Y., Xu H., Duan H., Ma P., Zhang L., Zamvil S.S. (2018). Vascular niche IL-6 induces alternative macrophage activation in glioblastoma through HIF-2α. Nat. Commun..

[100] Naschberger E., Liebl A., Schellerer V.S., Schütz M., Britzen-Laurent N., Kölbel P., Schaal U., Haep L., Regensburger D., Wittmann T. (2016). Matricellular protein SPARCL1 regulates tumor microenvironment-dependent endothelial cell heterogeneity in colorectal carcinoma. J. Clin. Investig..

[101] Ghajar C.M., Peinado H., Mori H., Matei I.R., Evason K.J., Brazier H., Almeida D., Koller A., Hajjar K.A., Stainier D.Y. (2013). The perivascular niche regulates breast tumour dormancy. Nat. Cell Biol..

[102] Hu H., Zhang H., Ge W., Liu X., Loera S., Chu P., Chen H., Peng J., Zhou L., Yu S. (2012). Secreted protein acidic and rich in cysteines-like 1 suppresses aggressiveness and predicts better survival in colorectal cancers. Clin. Cancer Res..

[103] Khan A.K., Wu F.T., Cruz-Munoz W., Kerbel R.S. (2021). Ang2 inhibitors and Tie2 activators: Potential therapeutics in perioperative treatment of early stage cancer. EMBO Mol. Med..

[104] Hendrikx S., Coso S., Prat-Luri B., Wetterwald L., Sabine A., Franco C.A., Nassiri S., Zangger N., Gerhardt H., Delorenzi M. (2019). Endothelial Calcineurin Signaling Restrains Metastatic Outgrowth by Regulating Bmp2. Cell Rep..

[105] Thomas H. (2016). Colorectal cancer: CRC endothelial regulation. Nat. Rev. Gastroenterol. Hepatol..

[106] Dirkx A.E.M., Egbrink M.G.A.O., E Kuijpers M.J., Van Der Niet S.T., Heijnen V.V.T., Steege J.C.A.B.-T., Wagstaff J., Griffioen A.W. (2003). Tumor angiogenesis modulates leukocyte-vessel wall interactions in vivo by reducing endothelial adhesion molecule expression. Cancer Res..

[107] Nambiar D.K., Aguilera T., Cao H., Kwok S., Kong C., Bloomstein J., Wang Z., Rangan V.S., Jiang D., von Eyben R. (2019). Galectin-1–driven T cell exclusion in the tumor endothelium promotes immunotherapy resistance. J. Clin. Investig..

[108] Motz G.T., Santoro S.P., Wang L.-P., Garrabrant T., Lastra R.R., Hagemann I.S., Lal P., Feldman M.D., Benencia F., Coukos G. (2014). Tumor endothelium FasL establishes a selective immune barrier promoting tolerance in tumors. Nat. Med..

[109] Streubel B., Chott A., Huber D., Exner M., Jäger U., Wagner O., Schwarzinger I. (2004). Lymphoma-Specific Genetic Aberrations in Microvascular Endothelial Cells in B-Cell Lymphomas. N. Engl. J. Med..

[110] Dunleavey J.M., Dudley A.C. (2012). Vascular Mimicry: Concepts and Implications for Anti-Angiogenic Therapy. Curr. Angiogenesis.

[111] Van der Schaft D.W., Seftor R.E., Seftor E.A., Hess A.R., Gruman L.M., Kirschmann D.A., Yokoyama Y., Griffioen A.W., Hendrix M.J. (2004). Effects of angiogenesis inhibitors on vascular network formation by human endothelial and melanoma cells. J. Natl. Cancer Inst..

[112] Zhao C., Gomez G.A., Zhao Y., Yang Y., Cao D., Lu J., Yang H., Lin S. (2018). ETV2 mediates endothelial transdifferentiation of glioblastoma. Signal Transduct. Target. Ther..

[113] Ricci-Vitiani L., Pallini R., Biffoni M., Todaro M., Invernici G., Cenci T., Maira G., Parati E.A., Stassi G., Larocca L.M. (2010). Tumor vascularization via endothelial differentiation of glioblastoma stem-like cells. Nature.

[114] Wang R., Chadalavada K., Wilshire J., Kowalik U., Hovinga K.E., Geber A., Fligelman B., Leversha M., Brennan C., Tabar V. (2010). Glioblastoma stem-like cells give rise to tumour endothelium. Nature.

[115] Akino T., Hida K., Hida Y., Tsuchiya K., Freedman D., Muraki C., Ohga N., Matsuda K., Akiyama K., Harabayashi T. (2009). Cytogenetic Abnormalities of Tumor-Associated Endothelial Cells in Human Malignant Tumors. Am. J. Pathol..

[116] Ohga N., Ishikawa S., Maishi N., Akiyama K., Hida Y., Kawamoto T., Sadamoto Y., Osawa T., Yamamoto K., Kondoh M. (2012). Heterogeneity of tumor endothelial cells: Comparison between tumor endothelial cells isolated from high- and low-metastatic tumors. Am. J. Pathol..

[117] Yan Y., Wei C.-L., Zhang W.-R., Cheng H.-P., Liu J. (2006). Cross-talk between calcium and reactive oxygen species signaling. Acta Pharmacol. Sin..

[118] Zheng Y., Zhou R., Cai J., Yang N., Wen Z., Zhang Z., Sun H., Huang G., Guan Y., Huang N. (2023). Matrix Stiffness Triggers Lipid Metabolic Cross-talk between Tumor and Stromal Cells to Mediate Bevacizumab Resistance in Colorectal Cancer Liver Metastases. Cancer Res..

[119] Sun Y., Wen F., Yan C., Su L., Luo J., Chi W., Zhang S. (2021). Mitophagy Protects the Retina Against Anti-Vascular Endothelial Growth Factor Therapy-Driven Hypoxia via Hypoxia-Inducible Factor-1α Signaling. Front. Cell Dev. Biol..

[120] Suleyman H., Kocaturk H., Bedir F., Turangezli O., Arslan R., Coban T., Altuner D. (2021). Effect of adenosine triphosphate, benidipine and their combinations on bevacizumab-induced kidney damage in rats. Adv. Clin. Exp. Med..

[121] Jackstadt R., van Hooff S.R., Leach J.D., Cortes-Lavaud X., Lohuis J.O., Ridgway R.A., Wouters V.M., Roper J., Kendall T.J., Roxburgh C.S. (2019). Epithelial NOTCH Signaling Rewires the Tumor Microenvironment of Colorectal Cancer to Drive Poor-Prognosis Subtypes and Metastasis. Cancer Cell.

[122] Varga J., Nicolas A., Petrocelli V., Pesic M., Mahmoud A., Michels B.E., Etlioglu E., Yepes D., Häupl B., Ziegler P.K. (2020). AKT-dependent NOTCH3 activation drives tumor progression in a model of mesenchymal colorectal cancer. J. Exp. Med..

